# Adolescents’ mental health concerns, reported with an idiographic assessment tool

**DOI:** 10.1186/s40359-020-00483-5

**Published:** 2020-11-07

**Authors:** Thomas Kristian Tollefsen, Sabrina Michelle Darrow, Simon-Peter Neumer, Turid Suzanne Berg-Nielsen

**Affiliations:** 1 Centre for Child and Adolescent Mental Health, Eastern and Southern Norway, Nydalen, P.B. 4623, 0405 Oslo, Norway; 2 Institute of Psychology, University of Oslo, Forskningsveien 3A, 0373 Oslo, Norway; 3 Department of Psychiatry, University of California San Francisco, 401 Parnassus Ave, San Francisco, CA 94143 USA; 4 Faculty of Medicine and Health Sciences, Norwegian University of Science and Technology, Klostergata 46, 7030 Trondheim, Norway

**Keywords:** Adolescent concerns, Adolescent mental health, Idiographic assessment, Counselling, Primary mental health care

## Abstract

**Background:**

Adolescents’ self-defined concerns about their mental health are understudied. Yet gaining insight into the individual concerns of this group could be helpful in providing better services to the adolescent population. In this study, an idiographic procedure called Assert was used to increase our knowledge of which concerns are reported by adolescents as the most salient, in a primary mental health care situation.

**Method:**

231 unique concerns were reported by 70 adolescents in a primary mental health context in Norway. These concerns were analysed qualitatively by a group of experts, to define categories. The distribution of these categories, and differences in gender and age, were analysed quantitatively. The alleviation experienced on the subjective concerns over the course of counselling was measured. Two linear multilevel models were analysed, to examine whether alleviation on self-defined concerns, as measured with Assert, differed-based on the main category of the concern or the number of times Assert was used.

**Results:**

Three main categories of concerns emerged, related to (1) Self, (2) Relationships and (3) Life domains; as well as nine sub-categories: (1a) Autonomy, (1b) Mental health, (1c) Somatic health, (2a) Improving of relationships, (2b) Feeling safe from people around them, (2c) Taking responsibility for others, (3a) School, (3b) Work and (3c) Spare time. Girls reported fewer Life domain concerns than boys. Younger adolescents (12–16) more frequently reported no Self concerns, and older adolescents (17–23) more frequently reported no Relationship concerns. The adolescents felt less bothered by their subjective concerns after counselling, and there were some differences in alleviation depending on the category of concern.

**Conclusions:**

The adolescents defined their own concerns at the start of counselling and were less troubled by these concerns after counselling. The content of the concerns might suggest that these adolescents experienced a need to improve across several arenas: personal, relational and academic. Research to extend the current study, to understand individual adolescent concerns, should include contextual and social factors and personal characteristics—and explore how counselling interventions can best help alleviate these personal concerns.

## Background

The Lancet Commission on Adolescent Health and Wellbeing has suggested that one way of ensuring adolescents receive higher quality mental health care is by giving them a stronger voice—both in the identification of health issues and in the development of appropriate solutions [1]. Yet research that identifies adolescents’ own definitions of their mental health issues is scarce [2], and little is known about the experiences of adolescents with mental health issues outside of treatment [3]. When examining the concerns adolescents have in their lives in general, some typical themes emerge—including school, peers, family and self and psychological well-being [4–8]—albeit with some gender differences, where girls tend to have a more holistic view of their concerns than boys [9]. New ways to help adolescents to express their concerns are needed and interventions should be based on the concerns presented by adolescents themselves [2].

For any mental health professional, accessing the subjective needs and concerns of their adolescent clients is a central task. Several studies have found that fear of stigma still hinders adolescents from disclosing mental health concerns [10–13] and that adolescents more often disclose this information to peers, siblings or on the internet [2]. Anttila et al. [2] also found that adolescents realised they needed help, and wanted to share negative experiences, yet often decided not to do so. Adolescents' diminished inclination to disclose information to adults may also reflect the adolescent developmental phase—wherein they generally tend to rely more on support from peers than from adults [14] and experience societal expectations to be more responsible for their own well-being [15].

### Mental health in adolescence

Adolescence is a period characterised by growth and development—across the physiological, cognitive, social and contextual domains [14]. However, controversies regarding the age-range of adolescence have existed for several decades. While most agree that adolescence starts at puberty, the endpoint is more debated. In this study, we have chosen to define an adolescent as an individual between 10 and 24 years. This is consistent with definitions by Laurence Steinberg [14]—a leading researcher on adolescence—and The Lancet Commission on Adolescent Health and Wellbeing [16].

Adolescence is recognised as a critical stage for the development of mental health, since 10–20% of children and adolescents are affected by mental health problems [17]. Several studies suggest that mental health in non-clinical adolescent populations, in high-income countries, has deteriorated over the last decades [16, 18–20]. It is suggested that this is due to an actual increase in symptoms and not merely to adolescents' willingness to report them [18, 20]. One possible reason for the increase in mental health complaints could be an increase in perceived stress and demands, especially in girls [21]. In line with this, Curran and Hill [22] found that perfectionism has increased in adolescents. The authors attribute this to several causes: social media, more ambitious and controlling parents and a more competitive society with unrealistic expectations. In addition, a report from The American Psychological Association [23] found that adolescent girls experienced sexualisation from an earlier age than previously, meaning that they experienced stricter body-ideals and that their self-image was closely connected to sexual attractiveness and behaviour. However, some researchers claim that the threshold for treating young adults for mental health issues has decreased, as they have found adolescents’ mean symptom level at start of treatment to diminish over an 8-year period [24].

Because mental health disorders tend to first occur in adolescence and increase the risk of disorders in adult life [25, 26], preventing the development of mental health disorders through early intervention in accessible and well-established primary health care for adolescents should be high-priority [16].

### Idiographic assessment

In primary mental health care services, counsellors need to make informed decisions as to whether an adolescent should receive short-term counselling in these services or be referred to specialised treatment. Thus, assessment is a vital part of this process. However—when the overall goal is to diagnose mental illness or measure effects of treatment [27], relying solely on standardised measures—the unique concerns of the adolescent may be overlooked [28]. To achieve a more individualised form of assessment, idiographic measures can be useful. Idiographic assessment is specifically adapted for each individual respondent [29], and can be used to collect information to assess the success of an intervention [30]. By systematically capturing adolescents’ subjective concerns, independent of other diagnostic procedures, idiographic assessment can be a supplement to the more standard therapeutic interview. This kind of assessment has gained popularity over the last decade but has not yet been systematically implemented in primary mental health services [31]. This is especially true with regard to children and adolescents. However, two assessment tools have recently stood out and have received attention: Top Problem Assessment (TPA) [32] and Psychological Outcome Profiles (PSYCHLOPS) [33]. Both are empirically validated. Yet neither has been implemented systematically in primary mental health care services for adolescents.

'Assert' is an idiographic measure developed for adolescents in primary mental health care, which has been implemented in Norway and evaluated in a randomised-controlled trial [34]. Assert was designed to increase adolescents’ involvement in and sense of control over the counselling they receive. This is achieved by letting adolescents define the concerns that are important to them and ensuring that said concerns are systematically addressed throughout the counselling. In Assert, the counsellor asks the adolescent client 'What matters to you'? And uses the adolescent’s concerns to guide their counselling. In every session, adolescent clients indicate—on a scale from 1 to 10—whether they are closer or further away from their self-defined concerns. A higher score indicates improvement, meaning that the adolescent is less bothered by this concern. As the concerns are revisited and scored in every session, it provides both the adolescent and the counsellor with feedback on their progress. Feedback-informed treatment may possibly have a positive effect on adolescents’ treatment outcomes [35]. However, no definitive conclusions can be drawn regarding the effectiveness of using feedback with child and adolescent populations, as the few published studies show inconsistent results [36].

Assert shares some qualities with other idiographic approaches. Yet the question 'What matters to you'?—rather than 'What’s the matter with you'?—demonstrates that Assert is founded on a salutogenic approach to mental health and well-being [37]. This differentiates Assert from idiographic approaches that focus on the adolescent’s problems. Allowing adolescent clients to change concerns, when they lose their relevance, also makes Assert dynamic and easy to adapt to the changing needs of an adolescent. This flexibility is enhanced by revisiting and scoring the concerns in every session, which helps the counsellor to maintain a steady focus on the concerns that are most relevant to the adolescent at the time.

In a randomised controlled trial (RCT) presented in an earlier paper, adolescents using Assert were compared with adolescents who received regular counselling [34]. Assert significantly reduced the adolescents’ external locus of control. This meant that they attributed less of their mental health improvement to external circumstances—particularly circumstances related to chance [34]. The intervention had no effect on mental health symptoms or quality of life. Yet a significant linear relationship was found between a strong external locus of control and experiencing more symptoms of mental ill-health and lower quality of life, before receiving Assert. This is in line with previous research, showing that the subjective experience of having control over the circumstances affecting one’s mental health is associated with better mental health outcomes (e.g. 38).

A qualitative study, in which counsellors who used Assert were interviewed, found that it was experienced as a positive way to gain insight into the youths’ concerns and needs [39]. Assert helped the counsellors to implement this focus in a methodized manner making it easier for the counsellors to systematically follow up the adolescents’ concerns over time. In addition, the counsellors experienced it as helping adolescents to express themselves and take responsibility for their own mental health, thus empowering them. The results indicate that Assert supports key characteristics of healthy adolescent development [40–42]. The effects of facilitating a continued focus on the adolescent’s perspective in counselling makes it possible to regard Assert as an integrated part of the counselling procedure, not just an assessment.

Adolescents are prone to instability in both their sense of self and their emotions [14, 43]—and possibly also in what they want to address in counselling. This means that what they deem important may change during counselling. When it applies flexible methods for assessing what matters to the adolescent, the focus of counselling can change with the client, thus indicating its utility for this age group. However—because of the unstandardised, heterogeneous and highly personalised nature of idiographic data—it can be a challenge to understand which concerns adolescents tend to believe are important. In order to use this to inform practice, the unique concerns presented by the adolescents need to be examined.

### Research questions

To use Assert to inform practice, it is essential to examine the concerns adolescents present, including whether: (1) there are similarities among adolescents’ concerns or they are mainly idiosyncratic; (2) concerns differ according to personal characteristics, such as age and gender; (3) the concerns were alleviated during counselling; (4) length of counselling impacts reported alleviation; and (5) there are differences in alleviation, based on the type of concern.

## Method

### Participants

#### Sample

This study is part of a larger clinical trial [34], where 150 adolescents were recruited. Only the 70 adolescents that comprised the intervention group, where Assert was used, are included in the sample for the current study. The 70 adolescents reported a total of 231 concerns with Assert. However, 12 did not report age or gender at T1 and were excluded from the descriptive analyses for T1. Of the 58 adolescents, 48 (82.8%) were female and 10 (17.2%) were male, with a mean age of 16.08 years (*SD* = 2.07). As described in a previous paper [34], the participants’ reported symptoms and problems with quality of life were in the sub-clinical range, as measured by the Norwegian version of the Inventory of Life Quality for Children and Adolescents [44] and the Norwegian version of the Strengths and Difficulties Questionnaire, Self-report [45].

### Procedure

The adolescents included had voluntarily contacted primary health services for psychosocial worries or concerns. These services receive adolescents between 12 and 23 years, and any adolescent within this age range, who presented with such concerns at a participating site, was consecutively recruited to the study by their counsellors in the first session. The services included in this study, do not provide counselling to individuals with severe psychopathological disorders or drug addiction. Regardless of this study, if a counsellor suspects or identifies severe psychopathology, the adolescent is referred to specialised services. Accordingly, adolescents with severe disorders, drug addiction or intellectual disabilities were not included in the study—which presupposed suitability for counselling in primary care with non-specialists. No data were collected from the adolescents that were referred to specialised care. All participating adolescents could enter a lottery to win a tablet or a headset. If the adolescents consented to participate, they received online questionnaires regarding their mental health and quality of life at the start of counselling and every fourth week, up to a maximum of five measurement points. Assert was administered and scored in each counselling session.

In Norway, primary mental health care services have a preventive focus, offering indicated preventive measures in the form of free short- to medium-term individual counselling for subjective psychosocial concerns, including light to moderate mental illness. These services are designed to be easily accessible and no referral is needed to get an appointment. There is a high degree of variability among the services, even among services organised within the same municipality. For longer and more intensive treatment, adolescents in all municipalities are referred to specialised services. Since the data used in this study was based on a larger effectiveness trial, it was crucial not to interfere with the ordinary procedures of the services; this would have had implications for the data. As no standardised procedures for assessment at the start of counselling are implemented in the services, no objective evaluation of the adolescents’ mental health status is conducted by the service providers. Furthermore, counselling is not standardised—counselling modalities range from informal, unstructured sessions to more structured interventions (e.g., cognitive behaviour therapy). If a counsellor participating in our study had training in a specific intervention, it was up to the counsellor to decide in which cases this was applied, since no formal instructions were given on recommended types of counselling and the type of intervention was not tracked. As the interventions are not standardised, there are also large variations in treatment length and in the frequency of the sessions. The counselling is provided by various health professionals—most commonly nurses, psychologists and social workers. The term 'counsellor' has been used to describe this group. The majority of counsellors participating in the study held specialised nursing degrees (50%), yet the group also included social workers (25%), clinical psychologists (15%) and some clinical psychology graduate students (10%). These factors add up to a group of services that are highly accessible, but also very heterogenic in terms of types of counselling, problem severity and counsellor background. A thorough investigation of all these factors was beyond the scope of this study.

### Measures

#### Assert

In the first session using Assert, the counsellor asks the adolescent, 'What matters to you'? The adolescent then defines up to three concerns they want to address during counselling, such as problems, goals and life domains. In this way, each adolescent will have their own, unique version of Assert. If an adolescent finds social situations challenging, for example, s/he might state: 'What matters most to me is to be able to be more active in social situations'. After the concern has been defined, the counsellor asks, 'On a scale from one to ten, where one is "not good at all" and ten is "very good", how do you feel about this concern'? In all subsequent sessions, the counsellor starts by asking, 'In the last session you said it was important to you to be more active in social situations. Does this still matter to you now'? If the adolescent answers 'yes', the concern is scored on the same scale [1–10]. If the concern is no longer important, the adolescent can replace or remove it.

### Analyses

#### Categorisation of concerns

The 70 participating adolescents reported 231 unique concerns in total. Given that adolescents can provide up to three concerns when using Assert and can change their concerns at each session, the number of concerns is higher than the number of included participants. To answer Research Question 1, the 231 concerns were presented anonymously to an expert group. This group consisted of two clinical child and adolescent psychologists, one child and adolescent psychiatrist and one social worker specialised in treating mental illness in children and adolescents. The group's members were highly experienced in the assessment and treatment of children and adolescents—as well as in professional development and education within the same field. They had limited knowledge of Assert and of the study, and none of the authors were included in the expert group.

An open, qualitative approach was used to extract the content of each concern, so that the most representative categories could be defined based on the available data. As the data consisted of short statements, it left less room for interpretation than do the longer texts that are more common in qualitative research. The group members first read and coded each concern independently of one another. Then, each group member's initial interpretation of the content of each concern was presented to the rest of the group. If the group members’ interpretations were consistent, they moved on to the next concern. If not, the concern was discussed until consensus around the content was reached. After the content of the concerns had been agreed upon, similarities between the content of each concern were identified and initial categories were defined. When all concerns had been placed into an initial category, the relationships between these initial categories were reviewed and the expert group identified broader categories into which these categories could be arranged, to create a hierarchical structure (i.e., the initial categories became sub-categories of the Self, Relationships and Life domains. See the Results section on page 14). Next, a revision of the hierarchy was performed, to examine whether each level of categorisation represented the concerns that were included. If not, the concern was moved to a more suitable category or the category was redefined. Finally, the group leader examined the results of the categorisation to identify any discrepancies or inconsistencies. In total, the whole group sat together for one and a half work-days to complete the categorisation.

#### Description of topics

Descriptive statistics were used to examine frequencies and distributions of concerns and categories. Chi-square tests were conducted to answer Research question 2, regarding differences in number of adolescent concerns within the three main categories, depending on gender and age. Fisher’s exact test was applied to accommodate for cell count below five [46]. SPSS ver. 23 [47] was used for the analyses.

#### Alleviation of concerns

To examine the alleviation of the adolescents’ concerns during counselling, the data was set up so that each line represented a concern identified and scored by an individual. The 58 participants reported 169 concerns. Concerns with missing post-test scores were excluded from the analyses. Seven participants changed one or more of their concerns during the course of counselling, causing missing post-test data on 34 (20.12%) concerns. Due to drop-out, another 11 (6.51%) concerns had missing post-test data. This left a total of 124 unique concerns. A linear multilevel model was analysed to examine whether the alleviation of concerns from start to end of counselling was significant and if this alleviation was present across all main- and sub-categories. Additionally, two linear multilevel models were analysed to examine whether an improvement on self-defined concerns—as measured with Assert—differed, based on the main category of the concern or the number of times Assert was used. 'Number of sessions where Assert was used' was applied as a proxy for the length of the counselling period, as specific data about the length of counselling was not available. Assert was used in every session the adolescents attended. The difference in the scores from the last to the first counselling session was used as the dependent variable. As each line of data represented a concern, not a participant, all models were nested by individual participant (random intercept). Least Significant Difference test was applied to adjust for multiple comparisons, the. SPSS ver. 23 [47] was used for all analyses.

## Results

### Categorisation of the concerns

A total of 231 unique concerns were reported with Assert, throughout the course of counselling. Four of the 231 concerns could not be categorised, and two other concerns were excluded to maintain participant anonymity. This left a total of 225 unique concerns. The analysis of these concerns yielded a hierarchical structure with three levels (Table 1). The highest level consisted of three broad categories: (1) Self, (2) Relationships and (3) Life domains. These three categories were further divided into sub-categories.

1. Self was divided into concerns regarding (1a) Autonomy (e.g. '*Dare to stand up for myself'*), (1b) Mental health (e.g. '*Limit rumination'*) and (1c) Somatic health (e.g. '*Better sleep'*).
2. Relationships were divided into concerns regarding (2a) Improvement of relationships (e.g. '*That the relationship between me, mom and dad improves'*), (2b) Need to feel safe from people around them (e.g. '*To not have to meet dad alone'*), and (2c) Taking responsibility for others (e.g. '*Be there for people who are not feeling good'*).
3. Life domains were further divided into concerns about (3a) School (e.g. *'Concentrate better in geography'*), (3b) Work (e.g. '*Get steady employment'*), and (3c) Spare time (e.g. '*I want to cook dinner at home'*).

**Table 1:** Main categories, sub-categories and dimensions derived from the unique concerns in Assert

Main category	Sub-category	Dimension
Self (*n* = 115)	Autonomy (*n* = 77)	Improve self-confidence/self-efficacy (*n* = 21)
		Become more self-assertive (*n* = 17)
		Be understood/believed/listened to (*n* = 15)
		Improve self-regulation/take care of myself (*n* = 15)
		Improve self-esteem (*n* = 6)
		Find/understand/explore identity (*n* = 3)
	Mental health (*n* = 27)	Anxiety/worrying (*n* = 10)
		Drugs/alcohol (*n* = 5)
		Rumination (*n* = 4)
		Depression/sadness (*n* = 3)
		Overcome negative experiences (*n* = 2)
		Body (*n* = 2)
		Self-harm (*n* = 1)
	Somatic health (*n* = 11)	Diet (*n* = 4)
		Exercise (*n* = 4)
		Pain (*n* = 2)
		Sleep (*n* = 1)
Relationships (*n* = 54)	Improve relationship to: (*n* = 38)	Family (*n* = 16)
		Friends (*n* = 14)
		Others (*n* = 7)
	Feel safe from: (*n* = 10)	Family (*n* = 7)
		Friends (*n* = 1)
		Others (*n* = 2)
	Take responsibility for: (*n* = 6)	Family (*n* = 3)
		Others (*n* = 3)
Life domains (*n* = 56)	School (*n* = 40)	Better performance (*n* = 25)
		Better wellbeing/motivation (*n* = 15)
	Spare time (*n* = 10)	Better performance (*n* = 7)
		Better wellbeing/motivation (*n* = 3)
	Work (*n* = 6)	Better performance (*n* = 5)
Total = 225	Total = 225	Total = 223, missing = 2

The lowest level of categorisation consisted of 30 different dimensions. Each of these were assigned to a sub-category. The dimensions are presented in Table 1.

Concerns about Self were most common (N = 115), whereas concerns about Relationships (N = 54) and Life domains (N = 56) were almost equally prevalent. The frequencies of the main categories, sub-categories, and dimensions are presented in Table 1.

### Distribution of concerns at T1

At T1, the majority of adolescents (45.6%) listed two concerns, 42.1% listed three and 12.3% had only one concern. Most adolescents (73.7%) reported one or more concerns about Self with Assert, where 40.4% had two concerns and 8.8% had one. 45.6% of the adolescents listed one or two concerns about Relationships, but no adolescent listed three Relationship concerns. Most adolescents (61.4%) reported no concerns about Life Domains. Among those who had concerns about Life domains, 33.3% had only one concern, 5.3% had two and none had three. The mean score on Assert at T1 was 3.62 (*SD* = 1.58) for concerns about Self, 4.33 (*SD* = 2.28) for Relationship concerns and 3.51 (*SD* = 1.69) for concerns about Life domains.

To further nuance the distribution of concerns, each participant’s combinations of categorised concerns were also examined. Around half (50.9%) had one or more concerns related to just one category. Seven combinations were found (Table 2). The largest single group comprised participants who only listed concerns about Self (33.3%); 73.6% listed one or more concerns about Self, either alone or in combination with other concerns. The frequencies of participants who reported Relationship concerns (45.6%) and concerns about Life domains (38.6%), were lower.

**Table 2:** Combinations of concerns observed

	Frequency	Percent
Self concerns only	19	33.3
Rel.^a^ concerns only	6	10.5
LD^b^ concerns only	4	7.0
Comb. incl. self and rel	10	17.5
Comb. incl. self and LD	8	14.0
Comb. incl. rel. and LD	5	8.8
Comb. incl. all cats	5	8.8
Total	57	100.0

^a^Relationships

^b^Life domains

A chi-square test was run, to examine differences in the number of concerns at T1 according to gender. There was a statistically significant difference between male and female adolescents in the number of Life domain concerns, at T1, χ^2^(2) = 6.199, *p* < 0.034. Girls reported fewer Life domain concerns (63.8% reported none) than boys (66.6% reported one or more). No other significant differences in gender were found in the number of concerns at T1.

To conduct the chi-square test using age, the age variable was split into early (12–16, *n* = 23) and late adolescence (17–23, *n* = 24). There was a statistically significant difference between early and late adolescents and the number of Self concerns at T1, χ^2^(3) = 8.813, *p* < 0.027. Participants in early adolescence more frequently reported no Self concerns (52.2%) than did participants in late adolescence (12.5%). There was a statistically significant difference between early and late adolescents and the number of Relationship concerns at T1, χ^2^(3) = 7.989, *p* < 0.013. Participants in late adolescence more frequently reported no Relationship concerns (75%), than participants in early adolescence (34.8%). No other significant differences in the number of concerns, depending on age, were found at T1.

### Alleviation of concerns during counselling

The analysis of the multilevel model showed that the concerns were significantly alleviated over the course of counselling [*F*(1, 121) = 153.564, *p* < 0.001], with a mean difference of 2.61, 95% CI [2.190, 3.023]. Significant alleviation was observed on concerns about Self [*F*(1, 68) = 123.166, *p* < 0.001], with a mean difference of 3.116, 95% CI [2.562, 3.670], Relationships [*F*(1, 27) = 19.771, *p* < 0.001], with a mean difference of − 1.517, 95% CI [0.731, 1.983] and Life domains [*F*(1, 24) = 25.350, *p* < 0.001], with a mean difference of 2.600, 95% CI [1.534, 3.666]. Significant alleviation was found on five of the nine sub-categories (see Table 3).

**Table 3:** Linear multilevel model showing alleviation from start to end of counselling divided by sub-categories

Sub-category	Numerator df	Denominator df	F	Sig
Autonomy	1	45.000	110.935	.000
Mental health	1	16	15.077	.001
Somatic health	1	5	10.292	.024
Improvement of relationships	1	22	17.041	.000
Taking responsibility for others	1	2	3.000	.225
School	1	18.000	15.346	.001
Spare time	1	3	6.943	.078

Dependent variable: score on Assert. Model could not be fitted for sub-categories ‘Work’ or ‘Need to feel safe from people around them’ because number of observations was less than or equal to number of model parameters

The results of the second linear multilevel model, nested by individual, indicated differences in the alleviation of concerns based on the main category [*F*(2, 118.125) = 3.595, *p* = 0.031]. The participants’ mean alleviation on concerns pertaining to Self (2.93, 95% CI [2.323, 3.544]) was significantly higher than on concerns about Relationships (1.63, 95% CI [0.775, 2.491]). The participants’ mean alleviation on concerns regarding Life domains (2.60, 95% CI [1.680, 3.529]), was not significantly different from mean alleviation on either category. Pairwise comparisons from the linear multilevel model are presented in Table 4.

**Table 4:** Pairwise comparisons of alleviation of concerns from the linear multilevel model

Main category	Main category	Mean difference	SE	*df*	Sig	95% CI	
Self	Relational	1.301	.486	117.731	.009	.337	2.264
	Life domains	.329	.520	118.980	.528	− .701	1.359
Relationships	Self	− 1.301	.486	117.731	.009	− 2.264	− .337
	Life domains	− .971	.593	116.004	.104	− 2.146	.203
Life domains	Self	− .329	.520	118.980	.528	− 1.359	.701
	Relational	.971	.593	116.004	.104	− .203	2.146

Dependent variable: difference between first and last score; adjustment for multiple comparisons: least significant difference

**Fig. 1:**
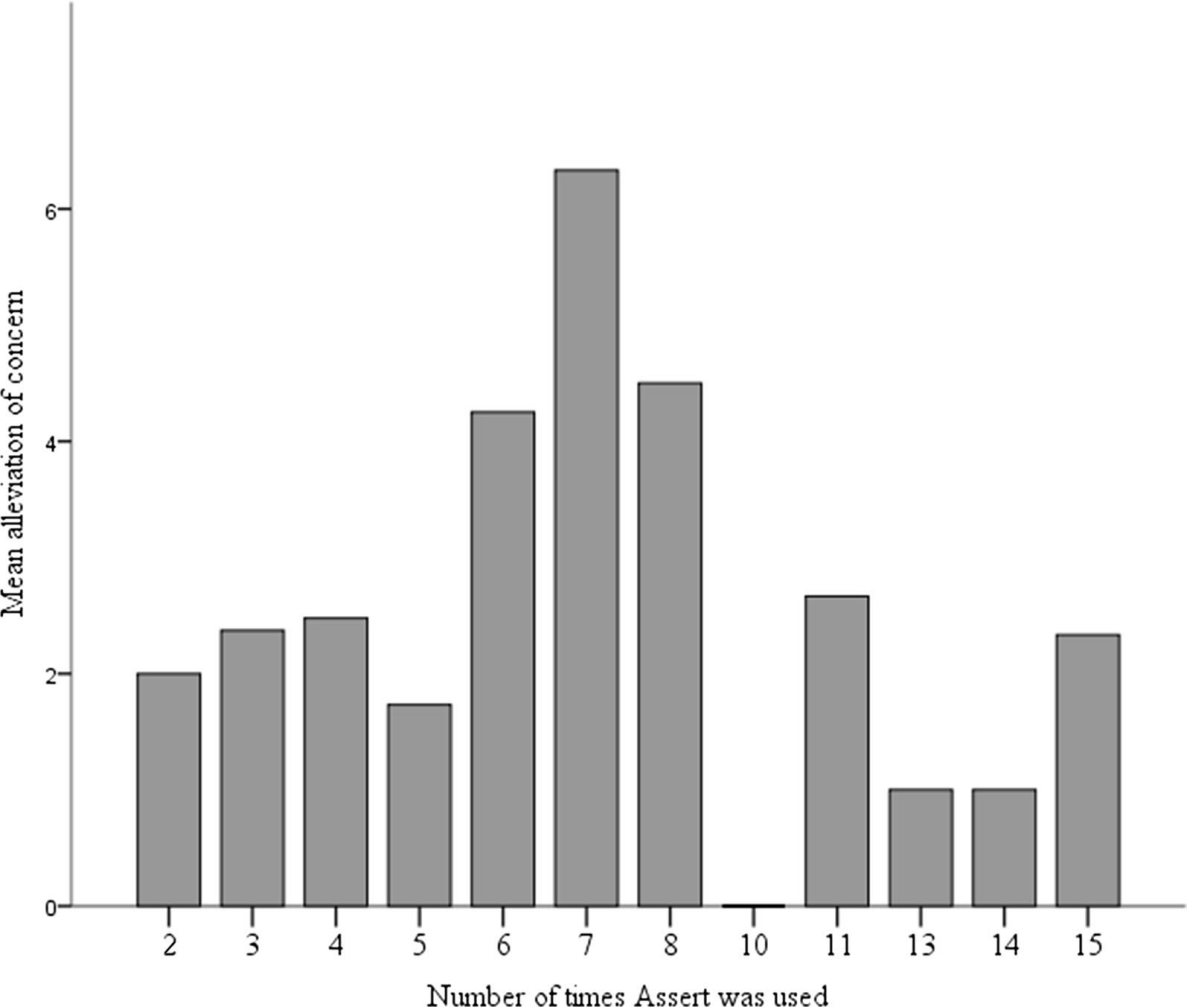
Mean alleviation of concerns by recurrence of Assert

The mean number of times Assert was used was 5 (*SD* = 3.27), the mode was 3 (*n* = 14) and the range was 2–15. No adolescent used Assert for either 9 or 12 sessions. A nested multilevel linear model was run, to examine whether the number of times Assert was used related to the amount of alleviation of concerns as reported by the adolescents. The model was significant [*F*(11, 83.915) = 2.401, *p* = 0.012]. The estimated effect of the number of uses of Assert showed a significantly higher mean alleviation of concerns when Assert was used in seven sessions [3.34, *t*(67.261) = 2.023, *p* = 0.047], compared with a recurrence of sessions of either less than seven or more than seven. Figure 1 shows the mean alleviation by the number of times Assert was used.

## Discussion

### Distribution and frequency of categories

In this study, Assert was implemented in a primary mental health service context that focuses on prevention, early intervention and counselling for less severe adolescent mental illnesses. Although we used an idiographic measure, there were similarities among the concerns presented by the adolescents.

Notably, adolescents most frequently reported concerns related to Self, followed by concerns about Life domains and, lastly, concerns about Relationships. Concerns relating to the sub-category Autonomy (under the main category Self) were clearly the most frequent. Within the main category Life domains, concerns about school recurred most often. Concerns about Improving relationships were the most frequent sub-category pertaining to Relationships. About one of four concerns relating to Self were sub-categorised as apparent mental health issues, equalling over one in ten of all concerns presented by the adolescents.

The high frequency of concerns within the sub-category Autonomy indicates adolescents’ need to be more in control of their lives and their desire to better regulate their emotions, assert themselves, trust in their capabilities and be more independent. As most standardised measures are designed to assess diagnostic criteria or symptoms of mental health impairment [27], however, it is reasonable to assume that these issues would not have been detected by other types of assessment that are commonly conducted in primary mental health care settings.

The relative low occurrence of concerns directly related to mental health provides evidence that the sample was sub-clinical, displaying mental health symptoms below the clinical cut-off, yet with a higher quality of life than outpatient populations [34]. However, the dimensions under the mental health sub-category show that certain internalising symptoms were prevalent, which is in compliance with other studies of similar samples (e.g. 48). Symptoms of internalising disorders often have their onset in adolescence, and early detection is a key to prevention. Even if their symptom levels did not warrant a diagnosis, all adolescents in this sample had a subjective need for help and chose to contact a counsellor. The adolescents were using their own words to describe issues impacting their wellbeing, and these words are not necessarily equivalent to the concepts professionals use to describe mental illness. Thus, independent of adolescents’ objective symptoms of mental illness, it would still be meaningful to measure the alleviation of subjective concerns that the adolescents decide are important for their wellbeing.

Most of the concerns around Relationships were about improving existing relationships; these concerns were similarly distributed between friends and family. It has been well documented that both family and peer relationships impact adolescent health and wellbeing (e.g. 49, 50). Concerns about Relationships may reflect that, during adolescence, individuals may begin to acknowledge that the quality of relationships is also their responsibility. For example, spending more time without their parents and changing schools pushes adolescents to take responsibility for maintaining—and sometimes redefining—old relationships, while seeking out and forming new ones.

Another noteworthy sub-category within the Relationship category was the need to feel safe around people the adolescents are close to. The majority of concerns in this area related to family relationships. Discovering potential violence or abuse at home is crucial to the security and wellbeing of the adolescent. Using idiographic measures to supplement therapeutic interviewing, in the detection and follow-up of these problems, could be beneficial. However, in the further assessment and handling of situations of this nature, one should not rely on idiographic measures alone, but should also implement appropriate efforts immediately.

Within the category Life Domains, concerns regarding School were the most frequent. Twenty-five of 40 school related concerns were about improving performance. Increased pressure to perform in school can be stressful, and stress and demands are related to mental and physical health complaints [21]. In addition, improving performance in the workplace—or in leisure activities like sports or keeping fit—was a frequently reported concern. The remaining concerns around Life domains were related to motivation and wellbeing, both of which are central to performance [51–53] and should therefore be seen in conjunction.

The results show that boys more frequently reported concerns about the Life domain than did girls. As this category includes concerns about School, it is possible that boys tend to have more difficulties with academic performance than girls [54, 55]. Admittedly, the sample includes a minority of males, so no firm conclusions should be drawn from these results. Moreover, age differences were found in a number of concerns related to Self and Relationships. Younger adolescents more often reported no concerns about Self, than did older adolescents. Older adolescents have a greater capacity for introspection and are more able to imagine their future identity and life choices—which could make them more concerned about who they are and who they will become [56, 57]. The opposite was seen for age differences in number of concerns around Relationships; older adolescents more often reported no concerns about Relationships than younger adolescents. Conflicts in the parent–child relationship may temporarily increase in early adolescence [58]. In addition, there are two school transitions for Norwegian adolescents in early adolescence. These often involve establishing new friendships and adapting to new social contexts. In contrast, older adolescents may have achieved more mature relationships with their parents and may have friendships that are more established. Still, since the analyses involved dividing a small sample into two groups, putting too much emphasis on these results could be misleading.

Some adolescent concerns were presumably not reported. For example, no adolescent reported concerns about social media, even though social media is widely important to many adolescents and has an influence on adolescents’ mental health (e.g. 59). One hypothesis could be that social media platforms in themselves do not feel very important to the individual adolescent—but rather the consequences of using them, such as a negative self-image [60, 61] or relationship concerns, which were reported by several adolescents in this study.

The relatively low level of concerns around Relationships, in this sample, is also noteworthy. Forming new relationships, both romantic and platonic—as well as renegotiating the relationship with one’s parents—is commonly assumed to be a large part of adolescence. In this sample, however, the adolescents reported two concerns about Self for every concern about Relationships. This could possibly be explained by the primary mental health care setting, which invites more concerns related to oneself and one’s wellbeing. Also, issues of self and identity are common among this age group [14]. The service setting could also explain why none of the adolescents reported larger issues, like worries about the environment or broader social or political concerns. It could be that the adolescents assumed that what mattered most to them had to be personal issues.

In summary, one could argue that a general tendency across all concerns is the wish for personal improvement, to feel better about oneself, to improve relationships and increase one’s school performance. This constant striving for improvement could potentially lead to feelings of insufficiency—that one is never good enough. Adolescence is a formative step in the human development, which is marked by leaps in social, cognitive and emotional capabilities, making it challenging to navigate through this phase. Despite increases in the adult population’s happiness and advances in health care services, the mental health of adolescents in the Western world has deteriorated over the last decades [16, 20]. Including youths' mental health concerns, as voiced by themselves, in low-threshold primary mental health care settings may contribute to limiting that deterioration.

### Alleviation of concerns during counselling

While Assert is designed to systematically capture what is important to adolescents at each session and allow for changes, the utility of Assert may be diminished if adolescents change all of their concerns each session. However, changes in concerns were only found in a minority of the participants. When the adolescents define what matters to them in their own words, they are presumably closer to the core of the problem than if a counsellor defined their concerns. This was also something the counsellors experienced while using Assert [39]. Measures used to assess and track progress in counselling need to be dynamic and develop along with the adolescent. After using Assert, adolescents experienced that their mental health was less dependent on external factors [34]. Defining their concerns in each session may have increased their sense of control over what was happening in counselling.

The multilevel models provide preliminary support that Assert could be sensitive to changes in self-defined concerns over the course of counselling. The score on each concern on Assert showed a significant alleviation of the burden or distress throughout counselling. This was true for all three main categories, as well as for five out of nine sub-categories. Concerns about Self were significantly more relieved during counselling than concerns about Relationships. The counselling was most often individual, and relational concerns may be more challenging to address in this service setting. Alleviating concerns about Self may be easier to navigate without involving other parties. Yet the scores on the adolescents’ concerns about their Relationships and Life domains also showed a significant alleviation from start to end of counselling. The adolescents may have managed to change their perceptions regarding their role in relationships or their efforts to improve—in school, for example. However, the absence of a comparison group in this study, means that no firm conclusions about the sensitivity of Assert can be made from this study.

The number of times that Assert was used had a significant effect on the alleviation of the adolescents’ self-defined concerns. This was particularly true when Assert was used in seven sessions; in these cases, the alleviation was greater than when it was used in either fewer or more sessions. These results may suggest that the adolescents were not in need of long-term treatment. Still, as less alleviation was achieved when Assert was used fewer than seven times, it also indicates that they did need a moderate amount of counselling.

The adolescents in this study all contacted a counsellor due to a subjective need for help. When they defined the concerns that mattered to them, and when these were addressed consistently over time, they felt less bothered by their concerns within relatively few counselling sessions. However, the variability was large, especially in the higher part of the range, possibly affecting the results.

### Limitations

The sample is eighty percent female. This is a common challenge in mental health research, as males are less prone to seek help for mental issues [62, 63]. Furthermore, the sample size is small, which also could limit generalisations about the adolescents’ concerns. Even though the number of participants was limited, the number of concerns that were used for the categorisation was fairly high. However, statistical analyses of smaller sub-categories and dimensions were challenging.

An obvious limitation was the lack of information regarding the counselling the adolescents received, as this could have shed light on differences in the alleviation of adolescent concerns according to the type of counselling received. Due to the high levels of variability between and within services—along with the study's naturalistic setting—it would have been too time-consuming and costly to collect the appropriate data to accommodate and control for the type of counselling, within the limits of this study.

Finally, it is possible that the alleviation of concerns could be due to regression towards the mean. Unfortunately, it is not possible to control for this with the available data, due to the design of this study. Thus, it cannot be ruled out that regression towards the mean accounted for at least some of the alleviation measured with Assert. Further, the aim of this study was not to examine the effect of Assert on alleviation of self-defined concerns, rather to examine if there was evidence that Assert was sensitive to changes in concerns over the course of counselling.

## Conclusions

The categorisation of concerns provides insight into what matters to adolescents who seek help from primary mental health services. The aim of helping adolescents to alleviate their burdens and concerns, before they develop into more serious disorders in the future, is at the core of services that focus on the prevention of mental illness.

Future research on the understanding of individual adolescents' concerns should include a larger and more gender-balanced sample to enable analyses of how contextual and social factors as well as personal characteristics might influence the content and distribution of the adolescents’ concerns. Moreover, more attention should be given to collecting data regarding the counselling provided, such as modality and length of counselling to better understand how to alleviate adolescents’ worries and concerns. Furthermore, more research is needed to examine whether this type of approach improves outcomes, such as counselling engagement and dropout, beyond a nomothetic approach. These suggestions would require significantly more research resources, considering the complex and heterogenous nature of most primary care services. Finally, future studies with Assert and mitigation of adolescent concerns about their psychosocial issues should include a comparison group which would allow controlling for a regression towards the mean.

## Data Availability

The datasets generated and analysed during the current study are not publicly available as consent for this is not obtained from participants. Publicising the data collected for this study is not approved by the Norwegian Regional Ethics Committee. However, data could be made available from the corresponding author on reasonable request and after approval from the Norwegian Regional Ethics Committee.

## Abbreviations

RCT: Randomised controlled trial.
TPA: Top Problem Assessment
PSYCHLOPS: Psychological Outcome Profiles
SPSS: The Statistical Package for the Social Sciences
